# Changes in local hemodynamic forces associated with arch branched endografts

**DOI:** 10.1016/j.jvssci.2026.100413

**Published:** 2026-02-24

**Authors:** William J. Yoon, Andres Schanzer, Kevin Mani, Anders Wanhainen

**Affiliations:** a Department of Surgical Sciences, Vascular Surgery, Uppsala University, Uppsala, Sweden; b Division of Vascular Surgery and Endovascular Therapy, University Hospitals Harrington Heart & Vascular Institute, Case Western Reserve University, Cleveland, OH; c Division of Vascular and Endovascular Surgery, University of Massachusetts Medical School, Worcester, MA; d Department of Diagnostics and Intervention, Vascular Surgery, Umeå University, Umeå, Sweden

**Keywords:** Branched endograft, Aortic arch aneurysm, Computational flow dynamics, Simulation

## Abstract

**Objective:**

Different zone 0 endograft designs have been introduced. This study sought to assess postimplantation hemodynamic changes induced by zone 0 endografts, with varied configuration of side branches, using computational models.

**Methods:**

Twenty-nine patients who underwent zone 0 endovascular repair with single-, double-, or triple-branched endografts (n = 7, n = 11, n = 11, respectively), using different configurations of antegrade (A) and retrograde (R) branches, were included. Computational simulations were used to assess postimplantation changes in peak flow rate, systolic blood pressure (SBP) and time-averaged wall shear stress (TAWSS) at the innominate artery (IA), right subclavian artery, right common carotid artery (CCA), left CCA (LCCA), left subclavian artery (LSA), and distal aortic arch, as well as the total displacement force (DF) of the endograft.

**Results:**

Regardless of orientation, IA side branch implantation increased the IA peak flow rate across all endograft designs, an effect significant only in single-branched devices (+70%, *P* = .02). This increase was accompanied by a significant decrease in peak flow in the LCCA and LSA for single- (*P* = .02 and *P* = .02, respectively) and double-branched devices (*P* = .01 and *P* < .001, respectively), whereas no such effect was observed with triple-branched devices. Notably, the two antegrade branches supplying the IA and LCCA and one retrograde branch to the LSA- and three retrograde branches (3R)-triple-branched designs had the opposite effect on LCCA blood flow (+8.3% vs −6.7% [*P* = .02], respectively), although their impact on LSA flow did not differ significantly (−7.5% vs −8.1% [*P* = .92], respectively). This resulted in disparate effects on distal arch flow (−0.3% vs +6.3% [*P* = .02], respectively). Postimplantation alteration in distal arch flow was progressively attenuated with more branches. The preimplantation to postimplantation SBP differences in the IA, LCCA, and LSA mirrored the corresponding changes in peak flow. Elevated peak flow in the IAs led to a significant postimplantation increase in the TAWSS across all device designs. In contrast, the LSA side branches with retrograde orientation in the two antegrade branches supplying the IA and LCCA and one retrograde branch to the LSA-triple-branched and 3R-triple-branched endografts demonstrated marked increases in TAWSS (36.1% vs 50.2%, respectively), despite there being no significant change in the mean flow rate. Although maximum DF varied between devices (single-branched, 32.8 N; double-branched, 23.9 N; 2A+1-triple-branched, 22.9 N; 3R-triple-branched, 28.9 N), a post hoc analysis showed that branch configuration did not significantly influence DF.

**Conclusions:**

The hemodynamic stability within the aortic arch improves with a greater number of endograft side branches. Retrograde branch orientation does not significantly affect flow rates or SBP in the supra-aortic vessels. Among the evaluated designs, the triple-retrograde-branched configuration demonstrated the most favorable flow characteristics.

**Clinical Relevance:**

Using patient-specific computational fluid dynamics simulations, this study compared the postimplantation local hemodynamic effects of single-, double-, and triple-branched endografts, using different configurations of antegrade and retrograde branches, in complex zone 0 aortic arch repair. Our study suggests two key findings: first, more branching improves hemodynamic stability by steadying blood flow, and second, the specific orientation of retrograde side branches does not affect the supra-aortic arteries. Properly validated, these findings may help to optimize branched endograft selection, ensuring stable hemodynamics for long-term patient success and device durability.

**Article Highlights**

• **Type of Research:** Human in vitro study
• **Key Findings:** Twenty-nine patients who underwent zone 0 endovascular repair using varied configurations of side branch endografts were analyzed using patient-specific computational flow simulations. The hemodynamic stability within the aortic arch improves with a greater number of endograft side branches. Retrograde branch orientation has no significant effect on the hemodynamics of the supra-aortic arteries.
• **Take Home Message:** Computational fluid dynamics provide patient-specific simulations to optimize branched endograft designs, refining complex aortic arch repair strategies.

The advent of branched endograft technology has broadened the scope of minimally invasive options for patients with complex aortic pathologies involving Ishimaru zone 0 who are considered unfit for traditional open repair. Different zone 0 endograft designs have been introduced, with varying numbers and orientations of the branches that accommodate the supra-aortic arteries.^1^ The introduction of these branched endografts poses several new challenges, chiefly regarding their impact on the local hemodynamics.

The placement of endografts in the aortic branches has been reported to alter flow patterns in the implanted region.^2^, ^3^, ^4^ Hemodynamic alteration have potential critical implications on perfusion, device durability, and, ultimately, patient outcomes. As such, understanding the hemodynamic implications of endograft implantation is crucial to determine the potential postintervention complications that may arise for the patient. Because these endografts are relatively new, there is limited understanding of how different branched endograft designs affect blood flow after zone 0 aortic repair. This remains a significant knowledge gap that may have critical implications on how this technology is adopted and disseminated.

Computational fluid dynamics (CFD) has increasingly been integrated into clinical practice. By generating three-dimensional (3D) models from patient-specific computed tomography data, CFD provides high-fidelity simulation and visualization of blood flow, facilitating the evaluation of postimplantation local hemodynamics after complex zone 0 arch repairs.^5^^,^^6^

The aim of this study was to assess the postimplantation hemodynamic changes induced by zone 0 endografts with a varied number and orientation of side branches using computational modeling analyses. Primary outcomes included near-wall hemodynamic related parameters including flow rate, pressure, and wall shear stress (WSS).

## Methods

### Patients and imaging data

This multicenter retrospective study included 29 patients who underwent zone 0 thoracic endovascular aortic repair (TEVAR) with single- (n = 7), double- (n = 11), and triple-branched (n = 11) endografts. Patients undergoing a single-branched TEVAR were treated with the Nexus Aortic Arch System (Endospan), which features an integrated branch for the innominate artery (IA), requiring two bypass connections between the right common carotid artery (CCA) and left CCA (LCCA), as well as the LCCA and left subclavian artery (LSA). The double-branch version of the Zenith Arch Branch (Cook Medical) with two bridging stents leading to the IA and LCCA was used for patients treated with double-branched TEVAR, and the triple-branch version was used for those who received triple-branched TEVAR. Double-branched TEVAR required revascularization of the LSA via a bypass from the LCCA. The debranching procedures were all staged and preceded endograft implantation. All double-branched endografts were designed with two antegrade branches for the IA and the LCCA. Among the 11 triple-branched endografts, 8 were configured with two antegrade branches supplying the IA and LCCA and 1 retrograde branch to the LSA (2A+1R configuration). The remaining three endografts were constructed with three retrograde branches (3R configuration).

All patients were deemed unfit for traditional open arch repair based on comorbidities and anatomical factors. All had high-resolution preoperative and postoperative computed tomography angiography imaging available for review. For each enrolled patient, anonymized DICOM datasets of the pretreatment and post-treatment computed tomography scans were obtained. This study protocol was approved by each institution's respective institutional review board. Owing to the retrospective nature of the study, the need for individual informed consent was waived.

### Patient-specific computational modeling

Computational modeling techniques were used to calculate patient-specific hemodynamics, based on the physical laws of fluid flows. Briefly, the methodology involved the 3D reconstruction of the aorta derived from imaging data, computational mesh generation, CFD simulations, all carried out with open source CFD modeling pipeline (SimVascular, Open Source Medical Software Corp).^7^ Postprocessing and analysis were performed using ParaView (Kitware), an open source software package devoted to data analysis and visualization.^8^

Preimplantion and postimplantation aortic geometric models were created by volume segmentation and 3D reconstruction of the aorta, based on anonymized computed tomography angiography scans of the 29 patients. The preimplantation geometric models included the entire ascending aorta, supra-aortic arteries emerging from the arch, and descending aorta. The postimplantation geometry incorporated the implanted device as well as any extra-anatomical bypasses.

Subsequently, each reconstructed geometry was discretized into tetrahedral finite element meshes, suitable for CFD simulation analysis, using the TetGen (Weierstrass Institute for Applied Analysis and Stochastics) generator within SimVascular.^9^ A mesh independence test for the simulations were carried out to identify the minimum grid resolution necessary to ensure simulation convergence.^10^ Based on these results, an 0.5-mm mesh size was applied in areas of interest, resulting in a mean of (5.61 ± 1.67) × 10^6^ elements.

### Computational details and boundary conditions

To accurately simulate the flow of blood within the model, the incompressible Navier-Stokes and continuity equations are applied to describe the conservation of momentum and mass, respectively.^11^ These equations were solved on the generated computational mesh, which is the discretized fluid domain. Owing to the lack of patient-specific inflow data, a physiological pulsatile velocity profile adapted from a previous study was imposed at the inlet.^12^ On the outlets, three-element Windkessel circuits were applied, representing the characteristic compliance and resistance of the downstream vasculature.^13^ Blood was characterized as an incompressible, laminar and Newtonian fluid with a constant density and viscosity of 1060 kg/m^3^ and 0.004 Pa s, respectively.^11^ The vessel walls were modeled as rigid. As such, a no-slip condition with zero velocity was specified at the aortic walls.^14^

CFD simulations were performed via an open source SimVascular software. All models were initialized using the results from the sixth cycle of a presimulation. The final models were then run with a time step of 1/1000 of a cardiac cycle, with data sampled at 50 points per cardiac cycle during the third cycle. Using 240 processors, the simulation was performed on the high-performance computing cluster at Case Western Reserve University.

### Hemodynamic outcome measures and statistics

The focus of this study was local hemodynamic response to the implanted device, focusing on localized flow pattern/rate, WWS, and hemodynamic displacement force (DF). The mean blood flow rate through the supra-aortic branches and descending aorta was calculated. WSS is related to the tangential force acting on the wall by the blood flow and plays a critical role in endothelial and platelet functions. Time-averaged WSS (TAWSS) is the average WWS over the cardiac cycle. Hemodynamic DFs acting on the surface of the endografts are due to pressure and friction exerted by the flow of blood on the vessel walls. DFs were calculated from postimplantation CFD simulation results, by taking an area integral of the net pressure and WSS over the surface of the endograft in systolic peak (DF = ∫p*d*A + ∫τ*d*A, where p = pressure, *τ* = WWS, and A = endograft surface area).^15^^,^^16^ Large DFs exerted on the endograft are the primary cause for distal migration of the device, which may further lead to a type I endoleak.^17^

Values were compared between preimplantation and postimplantation models using Wilcoxon signed-rank tests and between groups with different number of side branches using Wilcoxon rank-sum tests. Continuous data are reported as mean values with standard deviations. Differences between the preoperative and postoperative results are reported in percentages. A value of *P* value of <.05 was considered statistically significant.

## Results

### Supra-aortic branch blood flow

**Fig 1:**
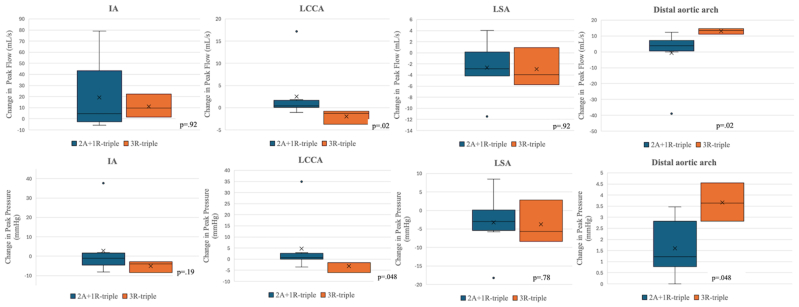
Relative postimplantation changes in branch hemodynamics stratified by combined (2A+1R)-triple-branched and all-retrograde (3R)-triple-branched patients. *IA*, innominate artery; *LCCA*, left common carotid artery; *LSA*, left subclavian artery; *2A+1R*, two anterograde branches for the IA and LCCA and one retrograde branch for the LSA; *3R*, three retrograde branches.

**Fig 2:**
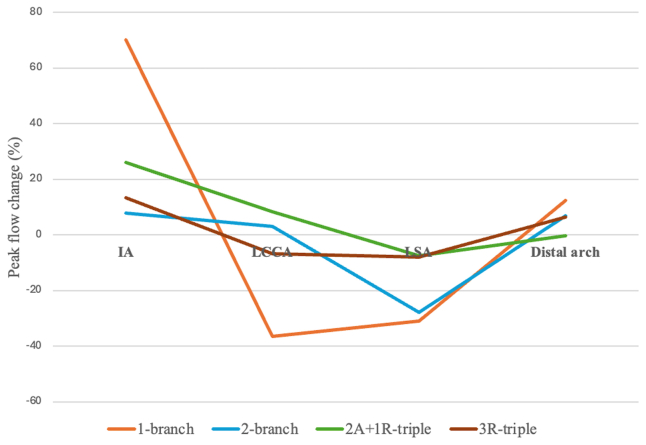
Line graphs comparing peak flow changes at four different blood flow domains. *IA*, innominate artery; *LCCA*, left common carotid artery; *LSA*, left subclavian artery; *2A+1R-triple*, two anterograde branches for the IA and LCCA and one retrograde branch for the LSA; *3R-triple*, three retrograde branches.

**Fig 3:**
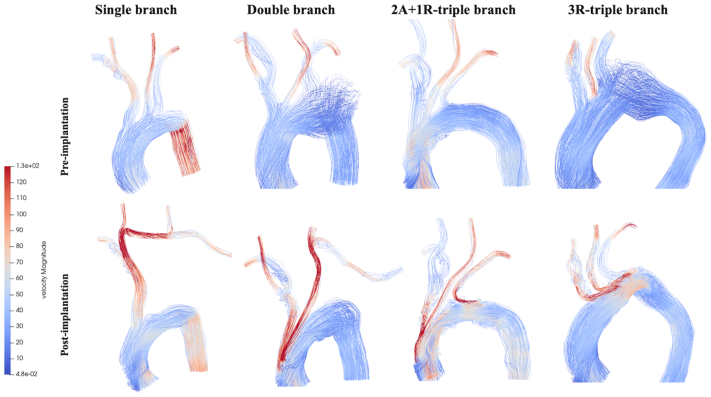
Colorimetric map showing streamlines with a flow velocity magnitude. 2A+1R-triple branch = 2 anterograde branches for IA and LCCA and one retrograde branch for the LSA; 3R-triple branch = 3 retrograde branches.

Table I presents preimplantation and postimplantation peak branch flow rates categorized by branch design. IA resulted in an increased postimplantation peak flow rate across all branch designs and regardless of its orientation. Single-branched endografts, which used the IA as the sole inflow vessel, led to a 70% increase in IA peak flow rate (*P* = .02). This observed increase was substantially higher than insignificant increases seen in double-, two antegrade branches supplying the IA and LCCA and one retrograde branch to the LSA (2A+1R)-triple-branched, and 3R-triple-branched devices. The increases in these devices were 7.8% (*P* = .12), 26% (*P* = .19), and 13.4% (*P* = .7), respectively. The increase in the IA flow rate influenced downstream blood flow, causing a significant postimplantation reduction in the flow rate in the LCCA and LSA for both single-branched (−36.6%, *P* = .02; −30.9%, *P* = .02, respectively) and double-branched (−9.3%, *P* = .01; −27.8%, *P* < .001, respectively) endografts. In triple-branched devices, there was no statistically significant difference in the LCCA or LSA flow rates post implantation vs pre implantation. Notably, the 2A+1R- and 3R-triple-branched designs produced opposite effects on the LCCA blood flow (+8.3% vs −6.7% [*P* = .02], respectively). However, their impact on the LSA flow did not differ significantly (−7.5% vs −8.1% [*P* = .92], respectively). Comparisons of postimplantation changes in the mean peak flow rates between two triple-branch designs are shown in Fig 1. Fig 2 highlights the fluctuation (Δ, %) in mean peak blood flow rates for four different endograft designs (single, double, 2A+1R, 3R) across four key blood flow domains (IA, LCCA, LSA, and distal arch). Preimplantion and postimplantation flow patterns are illustrated in Fig 3.

**Table I:** Peak flow rate (mL/s) of four endograft configurations

Branch design	Single (n = 7)	Double (n = 11)	Triple (2A+1R) (n = 8)	Triple (3R) (n = 3)
IA				
Pre implantation	69.6 ± 8.1	69.9 ± 4.5	73.8 ± 8.8	74.5 ± 15.8
Post implantation	118.4 ± 17.7	75.4 ± 8.4	92.9 ± 35.2	85.6 ± 26.1
Δ (%)	70.0	7.8	26.0	13.4
*P* value	**.02**	.12	.19	.7
LCCA				
Pre implantation	29.8 ± 1.7	28.2 ± 2.0	30.4 ± 1.0	29.2 ± 0.8
Post implantation	18.9 ± 3.4	25.6 ± 1.5	33.0 ± 6.5	27.2 ± 1.1
Δ (%)	−36.6	−9.3	8.3	−6.7
*P* value	**.02**	**.01**	.11	.2
LSA				
Pre implantation	34.2 ± 3.9	36.1 ± 2.8	35.5 ± 3.9	36.2 ± 1.2
Post implantation	23.6 ± 5.5	26.1 ± 2.2	32.8 ± 3.2	33.3 ± 3.5
Δ (%)	−30.9	−27.8	−7.5	−8.1
*P* value	**.02**	**<.001**	.16	.7
Distal arch				
Pre implantation	209.7 ± 9.3	209.1 ± 4.7	203.9 ± 6.9	206.9 ± 3.4
Post implantation	223.3 ± 14.9	223.3 ± 7.4	203.3 ± 18.9	219.9 ± 4.2
Δ (%)	12.3	6.8	−0.3	6.3
*P* value	**.02**	**.002**	.25	.1

*Δ (%),* indicates change between pre and post implantation; *IA,* innominate artery; *LCCA,* left common carotid artery; *LSA,* left subclavian artery; *2A+ R,* two antegrade branches supplying the IA and LCCA and one retrograde branch to the LSA; *3R,* all (IA, LCCA, LSA) retrograde branches.

Values are as mean ± standard deviation.

Boldface *P* values represent statistical significance. The *P* value reflects a comparison between pre implantation and post implantation; calculated *P* values obtained using Wilcoxon signed-rank tests.

### Supra-aortic branch pressure

Peak systolic blood pressure (SBP) was assessed, and values are presented in Table II. Increases in IA peak flow rate positively associated with postimplantation SBP changes across all antegrade branch designs; however, this increase was statistically significant only in the single-branch design (*P* = .03). For LCCA and LSA, decreased flow rates caused a significant decrease in SBPs in both single- (*P* = .02 [*P* = .02], respectively) and double-branched (*P* = .01, *P* ≤ .001) groups. In the triple-branched devices, there was no statistically significant postimplantation SBP change in the LCCA or LSA compared with the preimplantation baseline. Notably, owing to opposite effects on LCCA flow rate (antegrade vs retrograde orientation), the 2A+1R-triple- and 3R-triple-branch designs showed significantly different impact on the postimplantation SBP change (*P* = .048), with a 3.3% increase and a 2.2% decrease, respectively. Consistent with the similar LSA flow rates between the two triple designs, postimplantation LSA SBP change was not significantly different (−2.3% vs −2.6%; *P* = .78). Overall, the magnitude of postimplantation SBP change in the IA, LCCA, and LSA mirrored the corresponding postimplantation peak flow change.

**Table II:** Peak pressure (mm Hg) of four endograft configurations

Branch design	Single (n = 7)	Double (n = 11)	Triple (2A+1R) (n = 8)	Triple (3R) (n = 3)
IA				
Pre implantation	147.8 ± 2.6	147.2 ± 1.4	146.9 ± 1.6	146.3 ± 15.8
Post implantation	153.8 ± 5.0	147.5 ± 3.3	149.7 ± 14.6	141.2 ± 26.0
Δ (%)	4.1	0.2	1.9	−3.5
*P* value	**.03**	.58	.74	.1
LCCA				
Pre implantation	142.8 ± 3.2	139.6 ± 4.2	144.2 ± 1.5	142.1 ± 1.8
Post implantation	126.7 ± 19.6	132.7 ± 2.9	148.9 ± 12.8	139.0 ± 1.6
Δ (%)	−11.3	−5.0	3.3	−2.2
*P* value	.16	**.01**	.3	.2
LSA				
Pre implantation	139.6 ± 6.5	142.7 ± 4.3	141.3 ± 6.8	143.1 ± 1.6
Post implantation	119.9 ± 11.4	125.3 ± 4.4	138.1 ± 5.4	139.3 ± 5.8
Δ (%)	−14.1	−12.2	−2.3	−2.6
*P* value	**.02**	**<.001**	.3	.7
Distal arch				
Pre implantation	148.7 ± 2.9	148.5 ± 1.4	147.3 ± 2.0	147.8 ± 0.8
Post implantation	158.4 ± 5.3	153.6 ± 2.3	146.9 ± 5.8	151.4 ± 1.5
Δ (%)	6.5	3.4	−0.2	2.5
*P* value	**.02**	**.002**	.3	.1

*Δ (%),* change between pre and post implantation; *IA,* innominate artery; *LCCA,* left common carotid artery; *LSA,* left subclavian artery; *2A+1R,* two antegrade branches supplying the IA and LCCA and one retrograde branch to the LSA; *3R,* all (IA, LCCA, LSA) retrograde branches.

Values are mean ± standard deviation.

Boldface *P* values represent statistical significance. The *P* value reflects a comparison between pre implantation and post implantation; calculated *P* values obtained using Wilcoxon signed-rank tests.

### Distal arch flow and pressure

Relative to the preimplantation baseline, distal arch flow rates were significantly increased by 12.3% (*P* = .02) and 6.8% (*P* = .002) with the use of single- and double-branched endografts, respectively. Post implantation, neither the 2A+1R- nor the 3R-triple-branched configuration showed significant changes in distal arch flow rate compared with preimplantation baseline. Nevertheless, likely owing to distinct effects on LCCA and LSA flow rates, a significant difference in the change of peak flow rate was observed between the groups (*P* = .02): it decreased by 0.3% in the 2A+1R-triple branch group but increased by 6.3% in the 3R-triple branch group. Overall, the postimplantation alteration in distal arch flow rate was progressively attenuated with more side branches. The maximum distal arch flow rate Δ was highest in the single-branch, followed by double-branch, and lowest in the triple-branch endografts. The magnitude of postimplantation SBP change in the distal arch mirrored the corresponding postimplantation peak flow rate change.

### Branch WWS

The TAWSS at the endograft side branch was analyzed, with results categorized by device branch design in Table III. Mean TAWSS increases were observed across all IA branches post implantation, attributed to their anatomical position as the first aortic branch, resulting in accelerated flow: 79% in single (*P* = .02), 39.6% in double (*P* = .02), 60.9% in 2A+1R-triple (*P* = .02), and 94.9% in 3R-triple (*P* = .02). Reflecting increased peak flow rates in double-branched devices, LCCA TAWSS increased significantly by 56.5% (*P* = .03) with double-branched endografts. In contrast, postimplantation TAWSS changes were insignificant for both the 2A+1R- (1.7%, *P* = .95) and 3R- (−12.1%, *P* = .4) triple-branched endografts, with no significant difference in TAWSS change between the two designs (*P* = .28).

**Table III:** Time-averaged wall shear stress (TAWSS) (dynes/cm^2^) at side branches of four endograft configurations

Branch design	Single (n = 7)	Double (n = 11)	Triple (2A+1R) (n = 8)	Triple (3R) (n = 3)
IA				
Pre implantation	8.3 ± 2.3	8.6 ± 3.8	6.4 ± 3.1	7.9 ± 1.6
Post implantation	14.8 ± 4.1	12.1 ± 4.1	10.3 ± 2.2	15.4 ± 3.1
Δ (%)	79.0	39.6	60.9	94.9
*P* value	**.02**	**.02**	**.02**	**.02**
LCCA				
Pre implantation	N/A	16.3 ± 6.3	11.8 ± 3.2	17.4 ± 3.3
Post implantation	N/A	25.5 ± 6.8	12.0 ± 2.7	15.3 ± 3.0
Δ (%)	N/A	56.5	1.7%	−12.1
*P* value	N/A	**.03**	.95	.4
LSA				
Pre implantation	N/A	N/A	8.6 ± 3.5	7.5 ± 3.3
Post implantation	N/A	N/A	11.7 ± 3.7	11.2 ± 4.4
Δ (%)	N/A	N/A	36.1	49.3
*P* value	N/A	N/A	**.008**	.4

*Δ (%),* change between pre and post implantation; *IA,* innominate artery; *LCCA,* left common carotid artery; *LSA,* left subclavian artery; *2A+1R,* two antegrade branches supplying the IA and LCCA and one retrograde branch to the LSA; *3R,* all (IA, LCCA, LSA) retrograde branches.

TAWSS values were calculated at the side branch of the endograft. Values are mean ± standard deviation.

Boldface *P* values represent statistical significance. The *P* value reflects a comparison between preimplantation and postimplantation values. calculated *P* values obtained using Wilcoxon signed-rank tests.

The retrograde orientation of the LSA branch in both triple-branched designs led to a similar increase in postimplantation TAWSS (*P* = .38), with a 36.1% increase in the 2A+1R-triple group and a 49.3% increase in the 3R-triple group.

**Fig 4:**
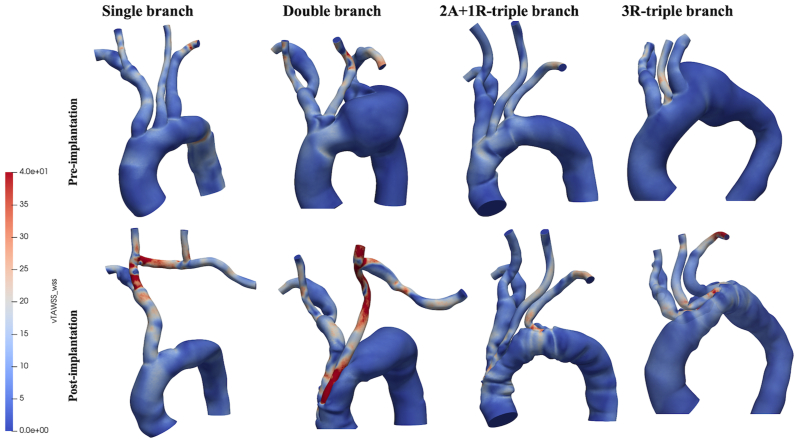
Colorimetric maps of time-averaged wall shear stress (TAWSS). *2A+1R-triple branch*, two anterograde branches for IA and LCCA and one retrograde branch for the LSA; *3R-triple branch*, three retrograde branches.

Fig 4 compares preimplantation and postimplantation TAWSS, showing that branched endograft inclusion increases both the magnitude and nonuniformity of TAWSS in the treated regions. High TAWSS regions occur at the origin of all side branches. With single-branched devices, localized areas of elevated TAWSS occurred in the branching zone, where the IA bifurcates into the right common carotid artery (CCA), as well as in the branching zone of the right CCA-LCCA bypass. Focal areas of increased TAWSS were also found at the anastomosis between the LCCA and the bypass in double-branched devices.

The implanted endograft is subjected to a DF resulting from the change in net momentum generated by the aortic blood flow. The mean maximum DF magnitude acting on the single-branched, double-branched, and 2A+1R-triple-branched and 3R-triple-branched devices was 32.8 N, 23.9 N, 22.9 N, and 28.9 N, respectively. The Kruskal-Wallis test did not find significant differences between the device branch designs, indicating that the DF is not significantly affected by the device branch design.

## Discussion

The use of arch branched endografts provides an effective solution for treating aortic arch pathologies by allowing vital flow to the supra-aortic arteries. The design of the branched endograft, specifically the number and orientation of branches, influences key hemodynamic parameters like blood flow rate, pressure, and WWSs, which in turn can impact long-term durability.

This study demonstrated that the placement of an IA side branch induces local flow acceleration, which directly impacts the downstream flow. This effect was most pronounced when a single-branched endograft was used. On analysis of postimplantation peak flow changes (%) at four different blood flow domains (namely, IA, LCCA, LSA, and distal aortic arch), it is clear that the number of endograft side branches affects local flow patterns. An increase in the number of side branches correlated with a decrease in postimplantation hemodynamic changes. This may be because the endograft morphology approaches that of the preimplantation aorta as the number of side branches increases.^18^

With triple-branched aortic arch repair, our results demonstrate how the orientation of the branch (ie, antegrade or retrograde) affects the blood flow. The IA flow rate increased using the 3R-triple-branched design, which was an unexpected outcome given the retrograde IA branch configuration. Compared with the 2A+1-triple-branch design (antegrade IA branch), this difference was smaller in magnitude and did not attain statistical significance. Conversely, although the flow rate was increased in 2A+1-triple-branched endografts featuring an antegrade-oriented LCCA branch, the flow rate was reduced when the LCCA branch was oriented retrogradely in 3R-triple-branch endografts. Hence, the elevated IA flow rate, regardless of branch orientation, is likely attributed to its anatomical position as the first branch of the aortic arch. These data also show a small, nonsignificant trend toward a decreased LSA peak flow rate change in both 3R and 2A+1R-triple-branched designs.

The choice between antegrade and retrograde branch orientation warrants further consideration. Recent studies indicate that early postprocedural stroke after branched aortic arch repair is closely linked to thrombus formation within the endograft branches.^19^ In addition to TAWSS, key hemodynamic parameters influencing branch thrombosis include flow velocities, and pressure changes.^20^ Idealized CFD models in previous reports suggest that antegrade branched grafts provide more favorable hemodynamics (higher flow and lower WWS) compared with the potentially stagnant retrograde configuration.^6^^,^^21^ However, our results indicate otherwise. Specifically, our CFD results (Fig 1) showed minimal differences in antegrade vs retrograde outflow rates and pressures for the IA, but significant differences for the LCCA when comparing 2R+1 A vs 3R models. Further, in our models, aortic and branch walls were assumed to be rigid and smooth, rendering them insusceptible to flow-induced damage. Consequently, our simulations cannot definitely determine which flow direction is more prone to branch thrombus formation. Future studies incorporating longer-term clinical data are necessary to evaluate how endograft branch orientation influences thrombosis risk after branched aortic arch repair.

In terms of supplying the systemic circulation, it is interesting to note that 3R-triple-branched design performed better than 2A+1R-triple-branched design in this study. Additionally, no statistically significant differences in peak pressure changes in the distal aortic arch were observed between the two triple branch configurations (Fig 1). Importantly, the current analysis suggests that the 3R-triple-branched device configuration does not have a negative impact on supra-aortic circulation and may even be the device design with the least significant changes in flow patterns across the aortic arch.

TAWSS describes the average magnitude of WSS averaged over the cardiac cycle. Fig 4 shows the influence of branch number on TAWSS distribution. Regions with very high TAWSS (>100 dyne/cm^2^) or extremely low TAWSS (<4 dyne/cm^2^)^22^—conditions known to be associated with an elevated risk of thrombus formation—were not observed in the study models. Regions of high TAWSS occurred at the origin of all side branches owing to abrupt changes in flow direction as well as in cross-sectional areas for the blood flow. High TAWSS was also found at the anastomosis between the bypass and the supra-aortic branches. The presence of high TAWSS values at these sites may indicate a risk of graft material fatigue, which could negatively impact long-term durability.^23^

In this respect, the use of multibranched endografts provides a compelling solution for total endovascular treatment, eliminating the complexities of surgical reconstruction for supra-aortic vessels and associated bypass-related complications. However, this may come at the cost of a greater risk of postoperative stroke, which is deemed to be associated with greater technical complexity and frequent catheter manipulation.^24^ Tazaki et al^25^ reported stroke rates of 7.8% with the single-branched, 33% with the double-branched and 42% for the triple-branched Inoue Stent Graft. In this context, single-branched devices could be better and easier to deploy, requiring less catheter manipulation in the aortic arch. Yet, in a recent meta-analysis, more proximal landing zones were associated with higher risks of stroke compared with distal (2.7% for zones ≥3, 6.6% for zone 2, 7.7% for zone 1, and 14.2% for zone 0).^26^ These findings underscore the urgent need to focus on the ongoing refinement of procedural methods to minimize such potential adverse outcomes.

Maintaining a stable position of the endograft is crucial for successful aortic arch repair. The implanted endograft in the aortic arch experiences a DF, the magnitude of which presents a risk to the long-term stability and integrity of modular endografts. This force results from the combined effect of the pressure and WSS generated by the pulsatile blood flow.^27^^,^^28^ Of these, blood pressure is several orders of magnitude greater (usually 10,000 times greater) than the shear stress. The maximum DF acting on the stent grafts ranged from 22.9 N to 32.8 N and a post hoc analysis indicated that the DF is not significantly affected by the device branch design. However, this study did not investigate how individual branched endograft characteristics—such as branch location, angulation, and size—influence DFs. Future work will analyze these factors to improve understanding of the correlation between branched endograft geometry and structural stability.

Although testing multiple configurations in a single anatomy via in silico modeling is a promising future direction, the current study utilized patient-specific models with generalized CFD boundary conditions to analyze endograft performance. This approach preserves critical geometric irregularities—such as tortuosity, aneurysmal shape, and branch angles—which fundamentally dictate local WSS and flow disruption. Additionally, these models capture realistic stent-vessel interactions often overlooked in idealized models.

## Limitations

The principal limitation of this study is that it relies solely on anatomical and imaging-based assessments, without corresponding data on clinical manifestations or outcomes. Given the relatively small magnitude of the hemodynamic changes observed, it is unlikely that any of the endograft configurations evaluated alter blood flow meaningfully enough to produce significant clinical effects. Additionally, the current study is limited in its design by the lack of consideration for preimplantation aortic morphology (eg, preoperative aneurysm size, aortic arch tortuosity, etc), which will undoubtedly affect the hemodynamic forces experienced by the endograft. Furthermore, endograft hemodynamic performance within the arch and its branches can be influenced by endograft and branch diameters, branch-to-main-graft angles, and branch-to-landing-zone angulation. Hence, future investigations will focus on these factors to evaluate long-term durability, moving beyond the baseline impacts of branch numbers analyzed here.

Additional limitations are inherent to this study design. The most significant of these is the small sample size. Still, the number of patients undergoing branched endovascular aortic arch repair is very limited, and this article focused on presenting the hemodynamic consequences of each endograft branching design. Nonetheless, because of the small sample size, we cannot rule out that the observed effects are the result of sampling error rather than a true effect of the designs.

Another important limitation is the lack of patient-specific boundary conditions, which may affect the accuracy and generalizability of the simulation results. Specifically, the use of a generalized pulsatile velocity waveform is a significant limitation. However, although the absolute values of the hemodynamic metrics might differ with patient-specific input, we believe the relative, comparative trends between different device configurations remain consistent. Furthermore, since the primary aim was to evaluate the impact of varying endograft side branch numbers on aortic hemodynamics, keeping the velocity waveform constant across all models eliminated the confounding effects of patient-specific heart rates and cardiac outputs, ensuring that variations in WSS and velocity were due solely to the device geometry.

Regarding the outflow boundaries, literature-based values for the three-element Windkessel circuit parameters were used. Although three-element Windkessel circuit parameters significantly influence outlet pressure and, by extension, flow distribution, using identical boundary conditions for all models ensures that the observed changes in hemodynamic results (eg, WSS, velocity, pressure) are attributable solely to the variations in the stent graft design, rather than being confounded by differences in distal compliance or resistance. This consistent approach minimizes confounding variables, allowing us to effectively isolate and understand the impact of the device design (ie, the number of side branches).

Finally, the simulations modeled the aortic wall as rigid, potentially overlooking the impact of aortic compliance on hemodynamics. Further improvements to the computational model can be achieved by incorporating wall distensibility and patient-specific conditions like inflow and outflow clinical data.

## Conclusions

Hemodynamic stability within the aortic arch improves with more endograft side branches, which mitigate flow rate fluctuations. Retrograde branch orientation does not significantly affect flow rates or SBP in the supra-aortic vessels. Of the designs evaluated, the triple-retrograde-branched configuration demonstrated the most favorable flow characteristics, suggesting significant potential for totally endovascular, patient-tailored zone 0 aortic arch repair.

## Author contributions

Conception and design: WY, AS, KM, AW

Analysis and interpretation: WY, AS, KM, AW

Data collection: WY, AS, KM, AW

Writing the article: WY

Critical revision of the article: WY, AS, KM, AW

Final approval of the article: WY, AS, KM, AW

Statistical analysis: WY

Obtained funding: Not applicable

Overall responsibility: WY

## Funding

None.

## Disclosures

W.Y. is a consultant for Cook Medical. A.S. reports consulting for Cook Medical, Artivion, and Philips (all compensation to the UMass Foundation).
